# Optogenetic stimulation of basal forebrain cholinergic neurons prevents neuroinflammation and neuropsychiatric manifestations in pristane induced lupus mice

**DOI:** 10.1186/s12993-023-00213-y

**Published:** 2023-06-15

**Authors:** Yang Yun, Xuejiao Wang, Jingyi Xu, Jingyu Chen, Xueru Wang, Pingting Yang, Ling Qin

**Affiliations:** 1 Department of Nephrology, Shengjing Hospital of China Medical University, Shenyang, China; 2 Department of Physiology, China Medical University, Shenyang, China; 3 Department of Rheumatology and Immunology, First Affiliated Hospital of China Medical University, Shenyang, China

**Keywords:** Basal forebrain, Cholinergic anti-inflammatory effect, Neuroinflammation, Neuropsychiatric lupus, Behavioral deficits, Optogenetics, α7nAChR

## Abstract

**Background:**

Neuroinflammation has been identified as one of the primary pathogenic factors of neuropsychiatric systemic lupus erythematosus (NPSLE). However, there are no dedicated treatments available in clinics to alleviate neuroinflammation in NPSLE. It has been proposed that stimulating basal forebrain (BF) cholinergic neurons may provide potent anti-inflammatory effects in several inflammatory diseases, but its potential role in NPSLE remains unexplored. This study aims to investigate whether and how stimulating BF cholinergic neurons has a protective effect on NPSLE.

**Results:**

Optogenetic stimulation of BF cholinergic neurons significantly ameliorated olfactory dysfunction and anxiety- and depression-like phenotype in pristane induced lupus (PIL) mice. The increased expression of adhesion molecules (P-selectin and vascular cell adhesion molecule-1 (VCAM-1)), leukocyte recruitment, blood-brain barrier (BBB) leakage were significantly decreased. Notably, the brain histopathological changes, including the elevated levels of pro-inflammatory cytokines (TNF-α, IL-6 and IL-1β), IgG deposition in the choroid plexus and lateral ventricle wall and lipofuscin accumulation in the cortical and hippocampal neurons, were also significantly attenuated. Furthermore, we confirmed the colocalization between the BF cholinergic projections and the cerebral vessels, and the expression of α7-nicotinic acetylcholine receptor (α7nAChR) on the cerebral vessels.

**Conclusion:**

Our data indicate that stimulation of BF cholinergic neurons could play a neuroprotective role in the brain through its cholinergic anti-inflammatory effects on cerebral vessels. Therefore, this may be a promising preventive target for NPSLE.

## Background

Systemic lupus erythematosus (SLE) is a chronic autoimmune disease characterized by the loss of immune tolerance to self-antigens, resulting in inflammation and severe end-organ damage. As one of the most potentially fatal manifestations of SLE, neuropsychiatric SLE (NPSLE) is a series of neuropsychiatric symptoms, manifesting as anxiety, depression, sociability deficits, cognitive impairment and seizures. As for the pathogenesis of NPSLE, it is generally believed that numerous inflammatory mediators from the peripheral circulation can disrupt the blood-brain barrier (BBB) and promote an inflammatory process in the central nervous system (CNS) causing glial activation, neuronal dysfunction and behavioral deficits [1, 2]. Therefore, targeting neuroinflammation may be a viable approach for improving the outcomes of NPSLE.

To date, the existing clinical drugs, such as corticosteroids and biologics, have not been demonstrated to have specific therapeutic effects for NPSLE patients. Glucocorticoid therapy provides immunosuppression, but comes with several adverse side effects like hyperlipidemia, hypertension, accelerated circulatory system diseases, diabetes, heightened susceptibility to infection and osteoporosis [3]. Additionally, psychiatric disorders can also be a consequence of corticosteroid administration in SLE patients [4]. The biologics, which typically target a single cytokine, have not been shown effectively in clinical trials, possibly due to the involvement of multiple immune cells and cytokines in the pathogenesis of NPSLE [5]. Thus, exploring new intervention strategies is essential.

The cholinergic system mainly originates from groups of cholinergic neurons within the basal forebrain (BF), that constitutes a major neuromodulatory system [6, 7]. Studies have demonstrated the significance of cholinergic anti-inflammatory mechanism in the regulation of pro-inflammatory mediators in sepsis, rheumatoid arthritis, multiple sclerosis and SLE[6, 8–10]. It has been noted that the dysfunction of the cholinergic anti-inflammatory system exists in SLE [11] and stimulation of cholinergic signals ultimately results in a decreased release of pro-inflammatory cytokines, consequently controlling inflammation and protecting against tissue injury [10, 12]. Thus, promptly and precisely regulating BF cholinergic neurons may be pivotal to strategies for combating NPSLE.

**Fig. 1:**
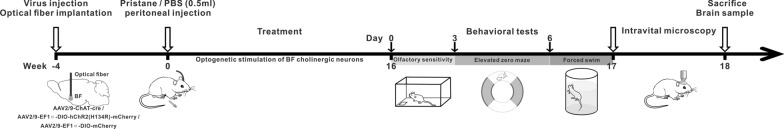
Experimental schedule Mice received microinjection of viruses and surgical implantation of optical fibers in the BF. Pristane or PBS was administered to mice via intraperitoneal injection. Optogenetic stimulation of BF cholinergic neurons was applied to mice for 4 months. Following a series of behavioral tests and intravital microscopy, mice were sacrificed and brain tissues were collected for further assays

The optogenetic technique, which controls neural activities through light stimulation, has been proven to be a valuable approach for investigating the mechanisms and therapeutic targets of neuropsychiatric diseases [13]. The pristane induced lupus (PIL) mouse model, despite its primary focus on replicating peripheral manifestations of SLE, has demonstrated some neurological and behavioral abnormalities resembling NPSLE [14]. Studies utilizing PIL mice have provided evidence of neuroinflammation in the CNS including overproduction of cytokines and chemokines, downregulation of hippocampal N-methyl-D-aspartate (NMDA) receptor subunits NR2A/2B, BBB leakage, immunoglobulin G (IgG) deposition, activation of glial cells (such as microglia and astrocytes) and lipofuscin accumulation within the brain [15–17]. Thus, the PIL mice are considered a valuable tool for studying NPSLE. Here, we aim to investigate whether optogenetically stimulating BF cholinergic neurons can potentially contribute to preventing neuroinflammation and subsequent brain pathology and behavioral deficits in PIL mice (Fig. 1). Our findings highlight that stimulation of BF cholinergic neurons exerts anti-inflammatory and neuroprotective effects on NPSLE, making it a potential intervention strategy for NPSLE in clinical settings.

## Results

### ***Optogenetic stimulation of BF cholinergic neurons ameliorated olfactory dysfunction and anxiety- and depression-like behaviors in PIL mice***

**Fig. 2:**
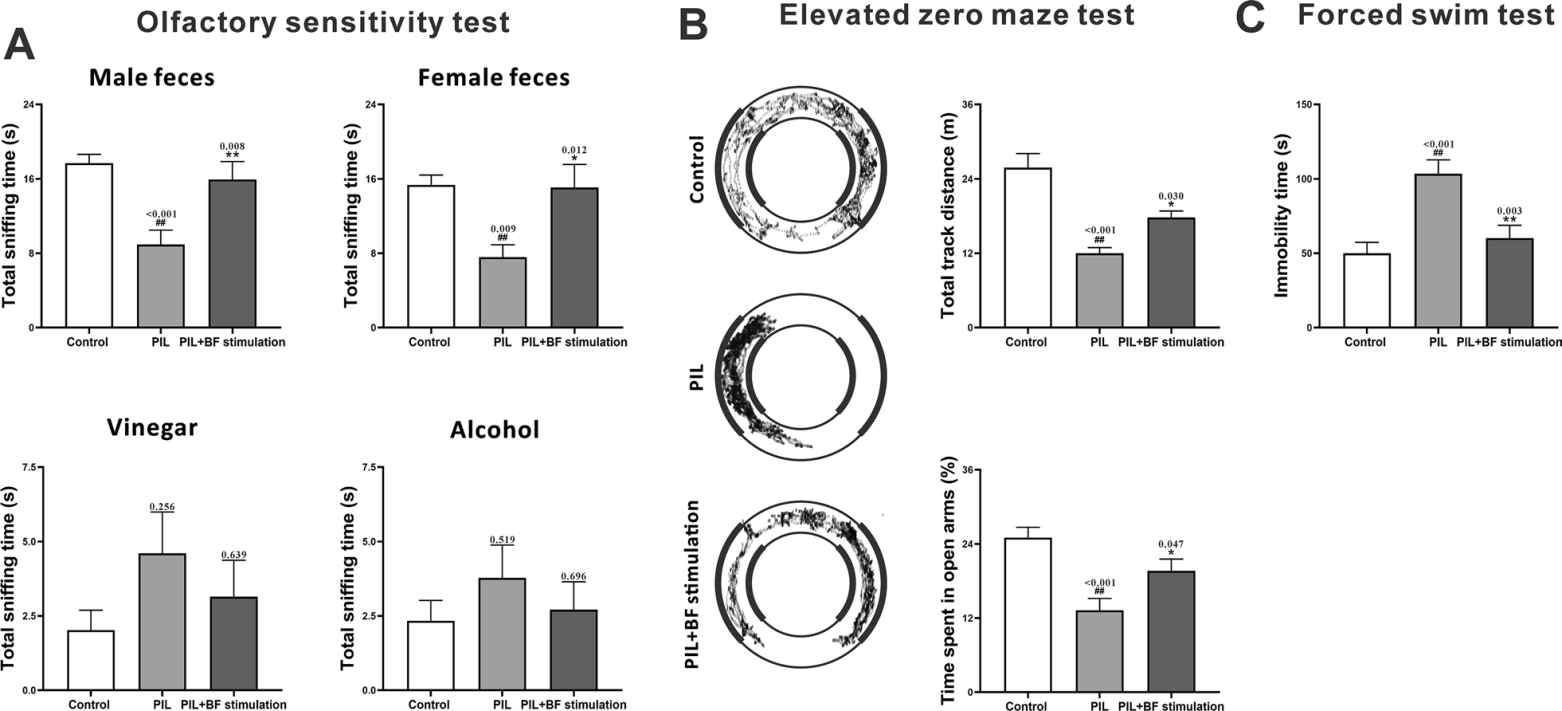
Effects of optogenetic stimulation of BF cholinergic neurons on behavioral deficits in PIL mice **A** Olfactory sensitivity test. Quantitative analysis of total time spent sniffing male feces, female feces, vinegar or alcohol. **B** Elevated zero maze test. Left panel showing representative track plots of the path. Right panel showing quantitative analysis of total track distance and percentage of time spent in the open arms (%). **C** Forced swim test. Quantitative analysis of immobility time. The data are expressed as the mean ± SEM (*n* = 12 in each group). One-way ANOVA followed by *Tukey’s post hoc* test: ^##^ *p* < 0.01 compared to the control group, ^*^ *p* < 0.05 or ^**^ *p* < 0.01 compared to the PIL group

To test the effect of optogenetic stimulation of BF cholinergic neurons on behavioral deficits, a series of behavioral tests were conducted in all tested groups. As shown in Fig. 2A, PIL mice spent less time sniffing male and female fecal odors (preferred odors) than controls in the olfactory sensitivity test. Following optogenetic stimulation of BF cholinergic neurons, the exploration time of the preferred odors in PIL mice improved significantly (Fig. 2A). Although not statistically significant, PIL mice spent a longer time sniffing vinegar and alcohol (aversive odors) compared to controls, and stimulation of BF showed a decreasing trend in the time spent on the aversive odors (Fig. 2A). The elevated zero maze test showed that PIL mice traversed significantly shorter total track distances and spent lower percentages of time in open arms than control mice, and stimulation of BF markedly reversed the anxiety-like behavior (Fig. 2B). Additionally, PIL mice exhibited longer immobility time than controls in the forced swim test, and stimulation of BF showed a remarkable reduction in immobility time (Fig. 2C). Collectively, these results indicate that stimulation of BF cholinergic neurons alleviated behavioral deficits in PIL mice, including olfactory dysfunction and anxiety- and depression-like behaviors.

### ***Optogenetic stimulation of BF cholinergic neurons attenuated endothelium activation, leukocyte recruitment and BBB leakage in PIL mice***

**Fig. 3:**
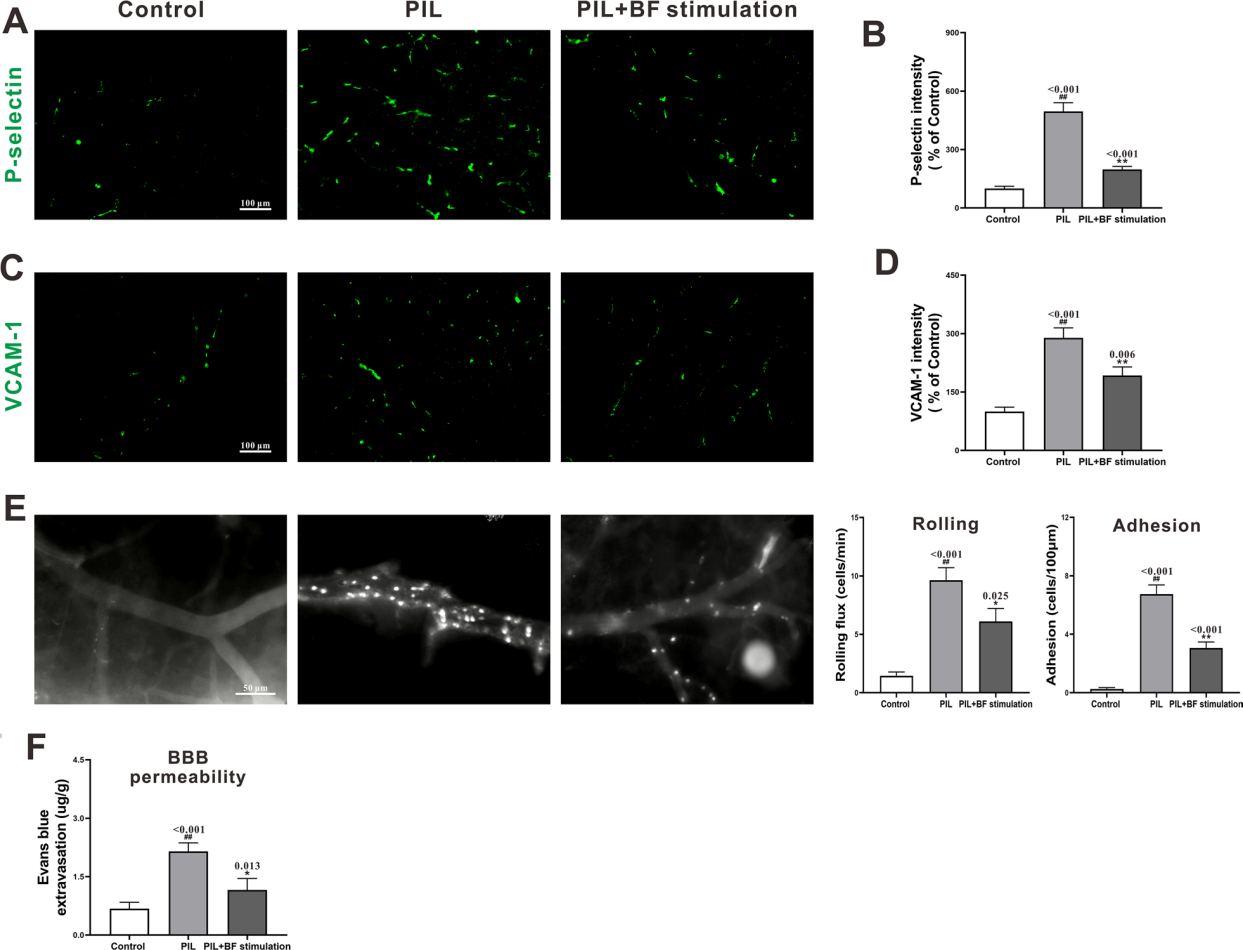
Effects of optogenetic stimulation of BF cholinergic neurons on endothelium activation, leukocyte recruitment and BBB leakage in PIL mice (**A**) and (**C**) Representative images of P-selectin and VCAM-1 expression. (**B**) and (**D**) Quantitative analysis of P-selectin and VCAM-1 expression by MFI and normalized to the control group. (**E**) Representative images and quantitative analysis of leukocyte rolling and adhesion. (**F**) Quantitative analysis of Evans blue dye extravasation. The data are expressed as the mean ± SEM (*n* = 12 in each group). One-way ANOVA followed by *Tukey’s post hoc* test: ^##^ *p* < 0.01 compared to the control group, ^*^ *p* < 0.05 or ^**^ *p* < 0.01 compared to the PIL group

Cerebral endothelium activation, characterized by increased adhesion molecule expression, is essential for circulating leukocyte recruitment and BBB impairment during neuroinflammation. To test the effect of optogenetic stimulation of BF cholinergic neurons on endothelium activation, we assessed P-selectin and vascular cell adhesion molecule-1 (VCAM-1) expression by immunofluorescence staining. As illustrated in Fig. 3A–D, compared to control mice with little expression of P-selectin and VCAM-1 in the cerebral vessels, PIL mice presented a significant elevation of these markers. Optogenetic stimulation of BF cholinergic neurons markedly decreased the elevated expression of P-selectin and VCAM-1 (Fig. 3A–D). We then utilized intravital microscopy to directly visualize leukocyte-endothelial cell interaction in cerebral vessels. Few rolling and adherent leukocytes were detected in control mice, while PIL mice had a considerable number of such cells (Fig. 3E). Stimulation of BF showed a significant reduction in leukocyte rolling and adhesion, which was concurrent with the alteration in adhesion molecule expression (Fig. 3A–E). Furthermore, significant BBB leakage was observed, as indicated by an increase in Evans blue content in brain tissues of PIL mice, but not in controls (Fig. 3F). After stimulation of BF, the extravasation of Evans blue decreased markedly (Fig. 3F).

### Optogenetic stimulation of BF cholinergic neurons decreased cytokine expression, IgG deposition and lipofuscin accumulation in the brain of PIL mice

**Fig. 4:**
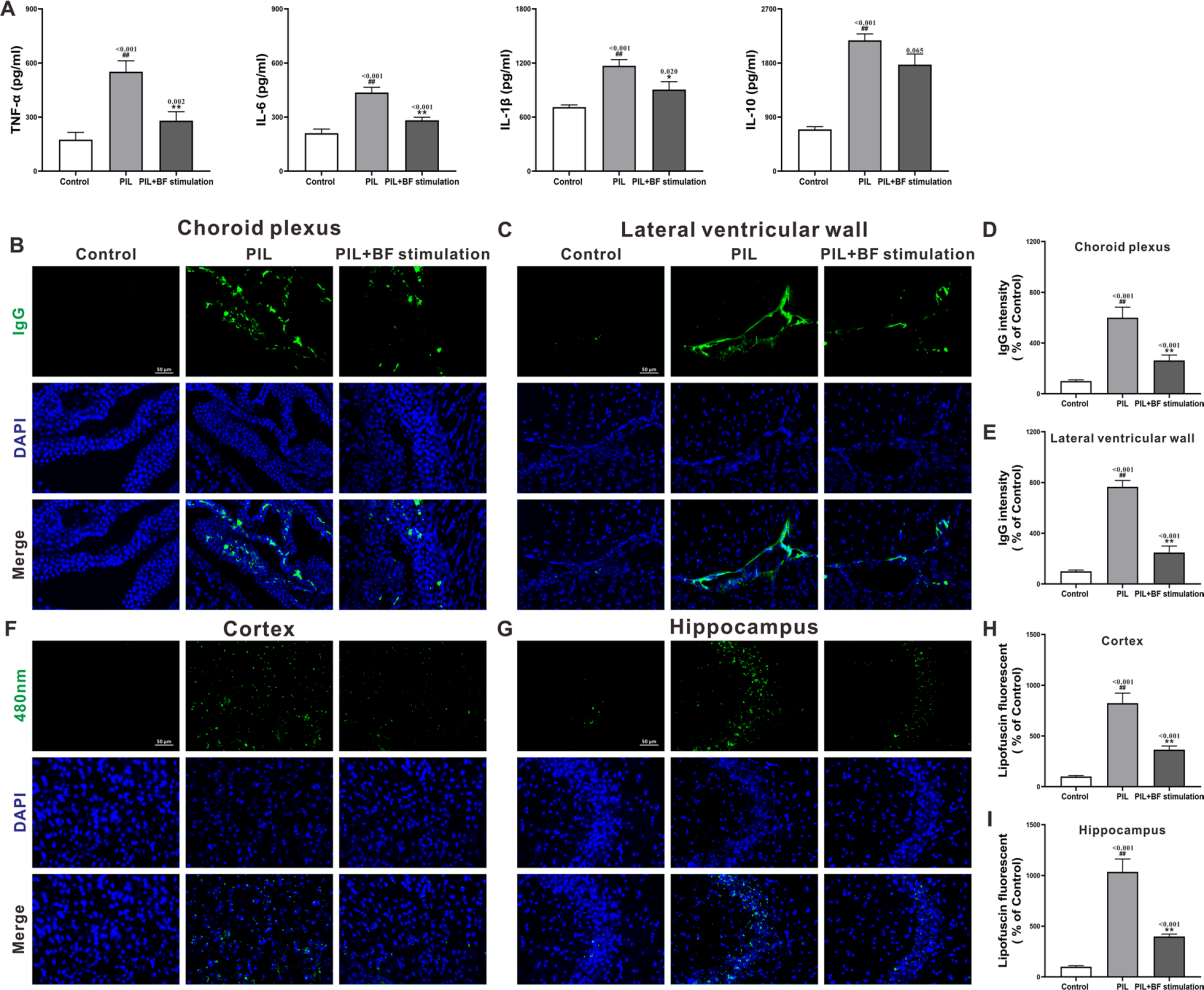
Effects of optogenetic stimulation of BF cholinergic neurons on cytokine expression, IgG deposition and lipofuscin accumulation in the brain of PIL mice **A** Quantitative analysis of the levels of brain cytokines (TNF-α, IL-6, IL-1β and IL-10). **B** and **C** Representative images of IgG deposition in the choroid plexus and lateral ventricular wall (green). DAPI staining for nuclei (blue). **D** and **E** Quantitative analysis of IgG deposition in the choroid plexus and lateral ventricular wall by MFI and normalized to the control group. **F** and **G** Representative images of autofluorescent lipofuscin at 480 nm exciting light (green) in the cortex and hippocampus. DAPI staining for nuclei (blue). **H** and **I** Quantification analysis of lipofuscin foci in the cortex and hippocampus and normalized to the control group. The data are expressed as the mean ± SEM (*n* = 12 in each group). One-way ANOVA followed by *Tukey’s post hoc* test: ^##^ *p* < 0.01 compared to the control group, ^*^ *p* < 0.05 or ^**^ *p* < 0.01 compared to the PIL group

To evaluate the effect of optogenetic stimulation of BF cholinergic neurons on brain pathophysiological changes, we examined cytokine expression, IgG deposition and lipofuscin accumulation in the brain by ELISA and immunofluorescence staining. Consistent with our previous results [15], PIL mice presented significantly increased expression of brain cytokines, including tumor necrosis factor-α (TNF-α), interleukin-6 (IL-6), IL-1β and IL-10 (Fig. 4A). Optogenetic stimulation of BF cholinergic neurons markedly suppressed the elevated expression of TNF-α, IL-6 and IL-1β, but not IL-10 (Fig. 4A). Through immunofluorescence staining, we observed a pronounced increase in IgG deposition in the choroid plexus and lateral ventricular wall of PIL mice compared to controls, and stimulation of BF dramatically rescued the heightened IgG deposition (Fig. 4B–E). Additionally, there was a notable rise in lipofuscin accumulation in the cortex and hippocampus of PIL mice compared to controls (Fig. 4F–I). Following stimulation of BF, lipofuscin accumulation decreased drastically (Fig. 4F–I). Thus, these results indicate that optogenetic stimulation of BF cholinergic neurons significantly alleviated inflammatory responses and tissue injuries in the brain of PIL mice.

### BF cholinergic projections colocalized with cerebral vessels and cerebral vessels expressed α7-nicotinic acetylcholine receptor (α7nAChR)

**Fig. 5:**
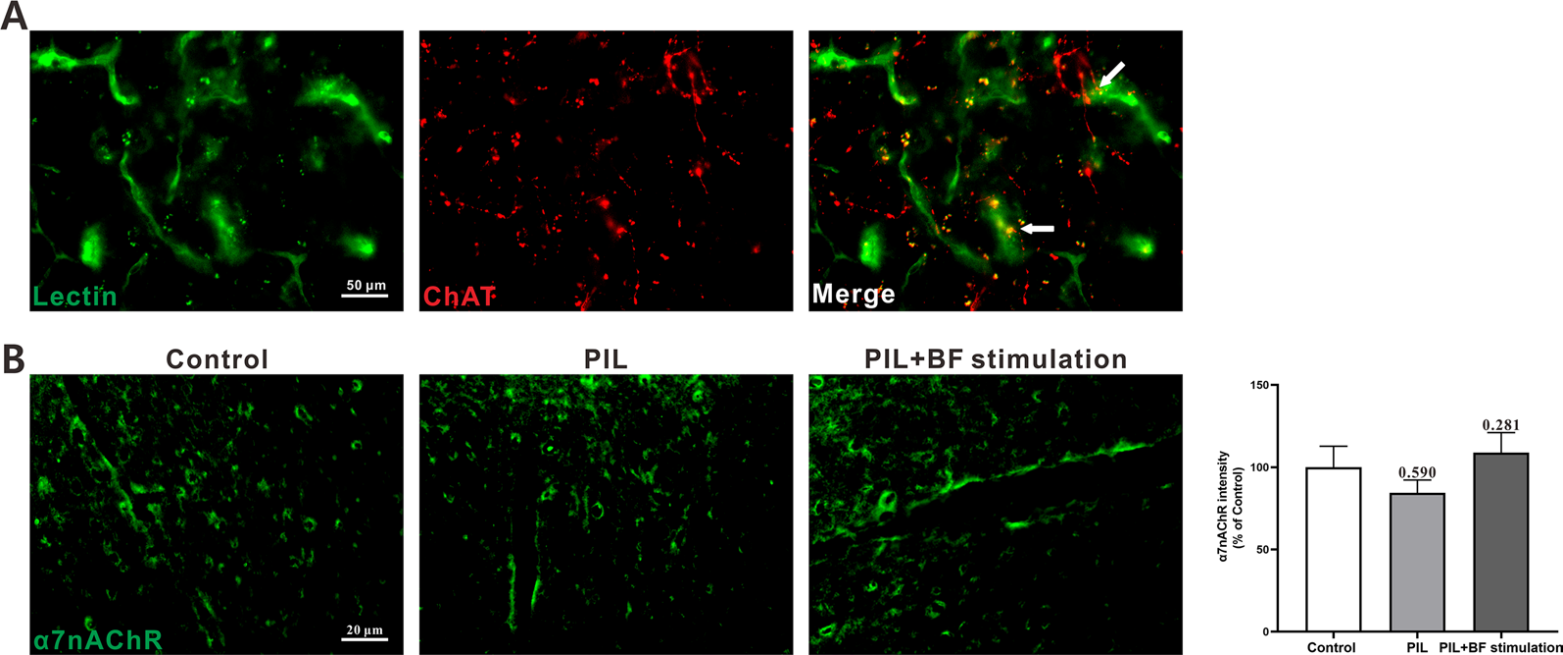
Examination for the relationship between BF cholinergic projection and cerebral vessels, and α7nAChR expression on cerebral vessels **A** Representative images of BF cholinergic projection (red) and lectin immunoreactive cerebral vessels (green). White arrow showing the co-localization between cholinergic projection (red) and cerebral vessels (green). **B** Representative images and quantitative analysis of α7nAChR expression in the cerebral cortex and normalized to the control group. The data are expressed as the mean ± SEM (n= 12 in each group). One-way ANOVA followed by Tukey’s post hoc test

To clarify the functional connection between BF cholinergic neurons and central inflammatory responses, central cholinergic circuits were investigated by immunofluorescence staining. Consistent with precious reports [18], we confirmed that some cholinergic projections colocalized with cerebral vessels, indicating that BF cholinergic neurons could modulate the function of cerebral vessels via cholinergic projections (Fig. 5A). Since α7nAChR has been reported to mediate the anti-inflammatory effects of cholinergic stimulation both in vitro and vivo [19], we evaluated whether α7nAChR was expressed on the cerebral vessels. Using immunofluorescence staining, we verified the expression of α7nAChR on the vessels in the cerebral cortex (Fig. 5B). No statistically significant differences were found in the expression of α7nAChR among all sampled groups (Fig. 5B). These results indicate that BF cholinergic neurons project to the cerebral vessels, which could provide a neuroanatomical basis for the anti-inflammatory effect of cholinergic signals on the cerebral vessels via α7nAChR.

## Discussion

The optogenetic technique utilizes light to control the activity of neurons which have been modified to express light-sensitive proteins, thus avoiding the nonspecific effects of electrical stimulation or pharmacologic manipulation [20]. Even without direct clinical use of optogenetics, optical control of neurons in animal models of human diseases enables a far more precise exploration of the pathogenesis and helps to address some clinically relevant problems. For example, first, via optogenetic intervention in mouse models of epilepsy, granule cells in the brain’s dentate gyrus were found to be implicated in the induction of seizures [21], and manipulation of neurons in regions distal to the seizure focus could serve as seizure-propagation “chokepoints” [22]. Second, optogenetic stimulation of cortical interneurons induced gamma oscillations, which are critical in attention and focusing, may provide the foundation for treatments of attention deficit hyperactivity disorder [23]. Third, optogenetic manipulation of phasic, but not tonic, firing in ventral tegmental area dopamine neurons of mice rapidly induced a depressive phenotype as measured by social avoidance and decreased sucrose preferenc [24]. Thus, optogenetic stimulation of BF cholinergic neurons in this study has potential clinical implications through revealing the pathogenesis of NPSLE and providing a new preventive target for NPSLE.

Interactions between the nervous and immune systems are central to the field of neuroimmunology. A major catalyst for growth in this field was the discovery that cholinergic signaling regulates immune functions and inflammatory responses [25]. The cholinergic system, through releasing acetylcholine (ACh), dominates systemic inflammation and inhibits pro-inflammatory cytokines production, depending on the α7nAChR [6]. The cholinergic anti-inflammatory mechanism is also under intensive investigations for autoimmune arthritis and SLE. Administration of α7nAChR agonists reduced clinical and pathologic signs of collagen-induced arthritis, and lowered serum levels of pro-inflammatory cytokines (i.e., TNF-α and IL-6) [26]. The severity of arthritis was exacerbated by deletion of α7nAChR [27]. Activation of the cholinergic anti-inflammatory system, either pharmacologically at the level of cholinergic receptors or upstream at the level of the vagus nerve, can protect the kidney by dampening inflammation and prevent the progression of hypertension in the setting of SLE [10–12, 28]. Additionally, recent findings have also characterized a role for central cholinergic signaling in controlling the immune responses and inflammation in the CNS [7]. The central cholinergic signaling mainly originates from the cholinergic neurons within the BF and that densely innervate the majority of cerebral vessels [29]. By immunofluorescence staining, we confirmed that the cholinergic projections from BF cholinergic neurons co-localized with the cerebral vessels (immunoreactive for lectin), providing the neuroanatomical basis for the regulatory role of cholinergic signals on the cerebral vessels (Fig. 5A). This result is consent with previous evidence showing the expression of α7nAChR by vascular endothelial cells [30–33]. Several in vitro studies reported that ACh and cholinergic agonists exert direct inhibitory effects on TNF-α-induced endothelial cell activation by blocking extracellular signal regulated kinase 1/2 (ERK1/2) and c-Jun N-terminal kinase (JNK) or nuclear factor-κB (NF-κB) signaling pathways via α7nAChR [19, 34]. In this report, we also confirmed the expression of α7nAChR on the cerebral vessels (Fig. 5B). Thus, optogenetic stimulation of BF cholinergic neurons may exert the anti-inflammatory effects on the cerebral vessels via α7nAChR.

The endothelium of cerebral vessels plays a pivotal role in maintaining BBB integrity and brain homeostasis. Exposure of endothelial cells to inflammatory stimuli activates intracellular signaling pathways resulting in the formation of multiple adhesion molecules, such as P-selectin and VCAM-1, and additional pro-inflammatory mediators [35]. These adhesion molecules facilitate the recruitment of circulating leukocytes and eventually lead to BBB leakage [30]. In this study, we found that optogenetic stimulation of BF cholinergic neurons decreased P-selectin and VCAM-1 elevation, leukocyte rolling and adhesion, and BBB leakage in PIL mice (Fig. 3). These ameliorations may be attributable to the anti-inflammatory effect of the cholinergic signals which inhibits the activation of endothelial cells, and subsequently decreases leukocyte recruitment and BBB impairment.

Another important finding of our study was the downregulated expression of elevated brain cytokines, decreased IgG deposition in the choroid plexus and lateral ventricle wall, and reduced lipofuscin deposits within neurons in the cortex and hippocampus in PIL mice (Fig. 4). The reduction of cytokines may be related to the lessened cytokine entries from peripheral circulation due to the improved BBB leakage, and the reduced local cytokine production following a decreased invasion of inflammatory stimulators. IgG deposition has been observed in the hippocampus of PIL mice [17], which may result from the impairments of BBB, blood-ventricular barrier and choroid plexus-vascular barrier [15]. Thus, the reduction of IgG deposition in the choroid plexus and lateral ventricular wall may be attributed to the improved vascular barrier function by BF stimulation. Lipofuscin, a product of metabolic imbalance of lipids and metals generally caused by neural oxidative stress, increases with age and pathological processes in CNS [36]. It has been suggested that lipofuscin participates in the pathogenesis of NPSLE in lupus-prone mice with depression [15, 37]. The decrease of lipofuscin deposits may be related to the downregulated expression of brain cytokines, which can induce a reduction in the synthesis of oxidative stress products within neurons. Stimulation of BF may decrease the cytokine-induced synthesis of oxidative stress products, consequently attenuating the generation and accumulation of lipofuscin.

Olfactory dysfunction, characterized by a decreased response to an olfactory stimulus, has been found in animal models of NPSLE such as anti-ribsomal P-injected mice, MRL/lpr mice and PIL mice [15, 38, 39]. Human patients with NPSLE were also reported to have a higher probability of olfactory abnormalities than healthy controls, associated with disease activity, age and positivity for anti-ribsomal P protein antibody [40]. The BF cholinergic system has been demonstrated to innervate the olfactory bulb and play a pivotal role in olfaction-mediated behaviors [41]. Stimulation of cholinergic system can elicit a wide-ranging enhancement of neural responsiveness to odorants and enhance animal performance in odor discrimination [42]. The strong modulation of the central cholinergic system on olfactory function may explain our present results showing that optogenetic stimulation of BF cholinergic neurons could ameliorate olfactory dysfunction in PIL mice (Fig. 2A). Affective deficits such as depression and anxiety are among the most common neuropsychiatric disturbances impacting quality of life within the NPSLE population. Our previous research has confirmed that PIL mice display anxiety- and depression-like phenotype [15]. Here, for the first time, we found that stimulation of BF could effectively rescue anxiety- and depression-like behaviors in PIL mice (Fig. 2B–C). Several studies have suggested the involvement of the central cholinergic system in anxiety- and depressive-like behaviors [43]. First, lesions of BF cholinergic neurons resulted in anxiety- and depressive-like behaviors [44]. Second, increasing the level of ACh in the brain could reduce anxiety [45]. Third, administering an acetylcholinesterase (the enzyme that degrades ACh) inhibitor into the brain could improve the anxiety-like behavior, as indicated by increased open arm exploration in the elevated plus maze test [46]. Notably, a recent study revealed that stimulation of cholinergic signals by systemic administration of α7nAChR ligands did not improve behavioral deficits in mice with advanced SLE [5]. This difference may be related to the different routes of treatment (direct stimulation of the CNS versus systemic drug administration).

In our study, the application of optogenetics in animal research effectively demonstrated that stimulating BF cholinergic neurons produces an anti-inflammatory effect within the CNS. However, a major obstacle to the application of this technology in humans is the concern of biological safety, specifically associated with the expression of artificially manipulated photosensitive opsins within neurons [47]. Therefore, addressing the priority of developing opsins that are safe, effective and compatible with human neural circuits is crucial. Nevertheless, a promising alternative approach is deep brain stimulation (DBS), which has been extensively explored in clinical treatments for neurodegenerative disorders such as Parkinson’s disease (PD) and Alzheimer’s disease (AD) with promising therapeutic outcomes [48, 49]. Currently, stimulating the BF region through DBS is an alternative approach for treating neuroinflammatory diseases in humans. In the future, the feasibility of optogenetic stimulation of BF in humans will increase with advancements in the development of safe photosensitive opsins.

## Conclusions

In summary, we present the first evidence showing that activation of BF cholinergic neurons may play a neuroprotective role in the brain through its anti-inflammatory effects on cerebral vessels. Our results highlight the significance of manipulating central cholinergic signals in attenuating behavioral deficits and reversing neuroinflammation in NPSLE, which may therefore be a potential preventive target for NPSLE. Further systemic experiments are warranted to examine whether and how stimulating BF cholinergic neurons can rescue the behavioral deficits and neural damage in NPSLE patients.

## Methods

### Animals

Specific pathogen-free BALB/c mice were purchased from Vital River Laboratory (Beijing, CHN). Female mice were used for the experiment at the age of 8 weeks. Animals were reared in standard animal cages under environmentally controlled laboratory conditions (12/12 h light/dark cycle, 22 ± 2 °C, 40–80% humidity) with *ad libitum* access to food and water. All efforts were made to minimize animal suffering. The animals were maintained and treated in compliance with the policies and procedures detailed in the “*Guide for the Care and Use of Laboratory Animals*” of the National Institutes of Health. The Animal Care and Use Committee of China Medical University reviewed and approved the animal experimental protocols and the treatment procedures (No. KT2018060).

### Experiment procedure

As shown in Fig. 1, mice were randomly divided into three groups (n = 12 per group): control, PIL and PIL + BF stimulation. At the initiation of the experiment, all mice were administered AAV2/9-ChAT-cre. Mice in the control and PIL groups were then given AAV2/9-EF1α-DIO-mCherry, while those in the PIL + BF stimulation group received AAV2/9-EF1α-DIO-hChR2(H134R)-mCherry. After recovery from microinjection of viruses and surgical implantation of optical fibers in right BF, each mouse received a single intraperitoneal injection of 0.5 ml phosphate buffer saline (PBS) or pristane (Sigma-Aldrich, St. Louis, MO, USA), respectively. Optogenetic stimulation of BF cholinergic neurons was administered to each mouse for 4 months following pristane or PBS injection. After behavioral tests and intravital microscopy recording, mice were sacrificed and brain tissues were harvested for further examinations.

### Stereotactic viral microinjection and optical fiber implantation to BF

Viruses (AAV2/9-ChAT-cre, AAV2/9-EF1α-DIO-hChR2 (H134R)-mCherry and AAV2/9-EF1α-DIO-mCherry) used in this study were obtained from Brain VTA (Wuhan, CHN). Cre-mediated recombination and channelrhodopsin2 (ChR2) expression were confined to choline acetyl transferase (ChAT) immunoreactive neurons located within the BF cholinergic neurons. The BF cholinergic neurons and their projections can be visualized by the fluorescence of mCherry. The activation of BF cholinergic neurons was selectively controlled by ChR2 with the presence of blue light stimulation. The surgical procedures of viral microinjection were as previously described [50]. Briefly, mice were anaesthetized and mounted onto a stereotaxic apparatus to adjust their skulls parallel to the reference panel. Then, AAV viruses were slowly injected into the right BF (anteroposterior, 0.1 mm; mediolateral, 1.5 mm; dorsoventral, 4.5 mm). An optical fiber was implanted directly above the viral injection site.

### Optogenetic stimulation

The optical fiber was coupled to a diode-pumped solid-state 473 nm laser and controlled by a laser driver. Light pulse trains (30 ms per pulse at 20 Hz for 15 s once per minute for 30 min) were controlled by a Master-8 pulse stimulator (Newdoon Inc., Hangzhou, CHN). Optogenetic stimulation of BF cholinergic neurons was conducted daily throughout the 4-month treatment period.

### Olfactory sensitivity test

The test used to assess olfactory sensitivity was conducted as previously described [51]. Each mouse was exposed to a piece of filter paper scented with an odorant for 2 min. Then, the scented filter paper was removed and the mouse was allowed to rest for 1 min. This procedure was repeated three times. Active investigation was defined as directed sniffing within 0.5 cm of the odorant source and the sniffing time was recorded. The total sniffing time of an odorant was obtained by summing the sniffing time for each trail.

### Elevated zero maze test

The elevated zero maze test was conducted as previously described [52]. Briefly, each mouse was placed at the open arm and allowed to conduct a 10-min free exploration. Total track distance and time spent in the open arms were digitally recorded, and then analyzed by custom-built programs. The percentage of time spent in the open arms was calculated using the following formula: [(time spent in the open arms)/(time spent in all arms) ×100].

### Forced swim test

Each mouse was placed into a glass beaker containing 3,000 ml of water (24 ± 1 °C) and allowed to habituate to swimming in it for 2 min. A 4-min test session was digitally recorded afterwards. With no way to escape, the mice began to struggle and swim, eventually displaying signs of behavioral despair, assessed as immobility[53]. Depression-like behavior was measured according to time spent immobile.

### Intravital microscopy in mouse brain

Intravital microscopy of the mouse cerebral vessels was performed as previously described [54]. Mice were mounted onto the stereotaxic apparatus and the optical fiber was carefully removed from the right skull. To create an optical clearing skull window for in vivo imaging, the left skull was thinned and treated with 10% Ethylene Diamine Tetraacetic Acid (EDTA) disodium (Sigma-Aldrich) for 5–10 min. Leukocytes were fluorescently labeled with rhodamine 6G (0.5 mg/kg body weight, Sigma-Aldrich). Rolling leukocytes were defined as leukocytes moving at a lower speed than erythrocytes. Adherent leukocytes were defined as cells that remained stationary for at least 30 s.

### Measurement of BBB permeability

The assessment of BBB permeability was performed by measuring Evans blue extravasation, as described previously [55]. Briefly, mice were administered 2% Evans blue dye solution (4 ml/kg, Beyotime biotechnology, Shanghai, CHN) intravenously 30 min before sacrifice. After the whole brains were harvested, brain tissues used for BBB permeability assessment were homogenized and incubated in formamide (24 h, 55 °C). The quantification of BBB permeability was performed by measuring the absorbance at 620 nm in the supernatant from each sample.

### ELISA for brain cytokine detection

Brain tissues, used for cytokine detection, were homogenized on ice in 0.01 M PBS (pH 7.4) with a protease inhibitor cocktail (diluted 1:100 (v/v), MedChemExpress, Shanghai, CHN), using a FastPrep-96 high-throughput homogenizer (MP Biomedicals, CA, USA) at 281.7 ✕*g* for 45 s (2 cycles). After homogenization, each sample was visually examined to ensure thorough sample disruption. Then, the samples were centrifuged at 12,000 rpm for 15 min at 4 °C to remove cell debris. The supernatants were collected and stored at − 80 °C until the measurement. The protein concentration was determined using a BCA protein concentration assay kit (Beyotime biotechnology). The cytokine levels in brain tissues were examinated using TNF-α, IL-6, IL-1β and IL-10 ELISA kits (Boster & Biological Technology, Wuhan, CHN). Experimental procedures were strictly followed according to the manufacturer’s instructions. Briefly, the protein samples were added to a 96-well microplate coated with antibodies against mouse TNF-α, IL-6, IL-1β, or IL-10. After 2 h incubation at room temperature, the microplate was incubated with horseradish peroxidase-conjugated streptavidin for an additional 1 h. The plates were then developed with tetramethylbenzidine substrate solution, and the optical densities were measured at 450 nm. Mouse cytokine standards with known concentrations were used to establish standard curves, and cytokine levels were expressed as relative titers.

### Immunofluorescence staining

Brain tissues used for immunofluorescence staining were fixed in paraformaldehyde, followed by immersion in sucrose solution. The tissues were cut into 10 µm-thick frozen sections and blocked with 10% goat serum. Then, sections were incubated with primary antibodies, including anti-P-selectin (1:80; Santa Cruz, CA, USA), VCAM-1 (1:200; Abcam, Cambridge, UK) and α7nAChR (1:100; ABclonal, Wuhan, CHN). On the following day, the primary antibodies were removed and sections were incubated with secondary antibody Alexa Fluor 488-conjugated goat anti-rabbit IgG (1:200, Proteintech, Wuhan, CHN). For IgG and lectin staining, sections were incubated with Alexa Fluor 488-conjugated goat anti-mouse IgG (1:200, Proteintech) and lectin solution (Thermo Fisher, Massachusetts, USA). DAPI staining (Beyotime biotechnology) was used to locate cell nuclei. To examine autofluorescent lipofuscin, regions of interest were captured at 480 nm exciting light. The mean fluorescence intensity of P-selectin, VCAM-1 and IgG and the mean gray value of autofluorescent lipofuscin were calculated using Image J software.

### Statistical analysis

GraphPad Prism V8 software (La Jolla, CA, USA) was used for statistical analysis. Before applying parametric statistics, all data were assessed for normality using the *D’Agostino-Pearson omnibus normality* test. Data were expressed as the mean ± SEM. Differences in this study were analyzed using one-way analysis of variance (ANOVA) followed by *Tukey’s post hoc* test for multiple comparison. *P* < 0.05 was considered a statistically significant difference in all tested groups.

## Data Availability

The data are available for any scientific use with kind permission.

## Abbreviations

NPSLE: Neuropsychiatric systemic lupus erythematosus
BF: Basal forebrain
PIL: Pristane induced lupus
VCAM-1: Vascular cell adhesion molecule-1
BBB: Blood-brain barrier
α7nAChR: α7-nicotinic acetylcholine receptor
SLE: Systemic lupus erythematosus
NMDA: N-methyl-D-aspartate
IgG: Immunoglobulin G
CNS: Central nervous system
ACh: Acetylcholine
ERK1/2: Extracellular signal regulated kinase 1/2
JNK: c-Jun N-terminal kinase
NF-κB: Nuclear factor-κB
DBS: Deep brain stimulation
PD: Parkinson's disease
AD: Alzheimer's disease
ChR2: Channelrhodopsin2
ChAT: Choline acetyl transferase
PBS: Phosphate buffer saline
EDTA: Ethylene diamine tetraacetic acid
TNF-α: Tumor necrosis factor-α
IL-6: Interleukin-6
ANOVA: Analysis of variance
